# Prolonged Scar-in-a-Jar: an in vitro screening tool for anti-fibrotic therapies using biomarkers of extracellular matrix synthesis

**DOI:** 10.1186/s12931-020-01369-1

**Published:** 2020-05-07

**Authors:** Sarah Rank Rønnow, Rand Qais Dabbagh, Federica Genovese, Carmel B. Nanthakumar, Vikki J. Barrett, Robert B. Good, Sarah Brockbank, Simon Cruwys, Henrik Jessen, Grith Lykke Sorensen, Morten Asser Karsdal, Diana Julie Leeming, Jannie Marie Bülow Sand

**Affiliations:** 1 Nordic Bioscience A/S, Herlev, Herlev Hovedgade 205-207, DK-2730 Herlev, Denmark; 2 Department of Cancer and Inflammation Research, Institute of Molecular Medicine, University of Southern Denmark, Odense, Denmark; 3 Department of Fibrosis DPU, Respiratory TA, GlaxoSmithKline, Stevenage, UK; 4 Innovative Medicines Unit, Grünenthal Innovation, Aachen, Germany; 5 Present Address: Medicines Discovery Catapult, Alderley Edge, Cheshire, UK; 6 Present Address: TherapeutAix AG, Aachen, Germany

**Keywords:** Scar-in-a-jar, Fibrogenesis, IPF, Fibroblasts, Collagens, Extracellular matrix, Fibrosis, Drug development, In vitro

## Abstract

**Background:**

Idiopathic pulmonary fibrosis (IPF) is a rapidly progressing disease with challenging management. To find novel effective therapies, better preclinical models are needed for the screening of anti-fibrotic compounds. Activated fibroblasts drive fibrogenesis and are the main cells responsible for the accumulation of extracellular matrix (ECM). Here, a prolonged Scar-in-a-Jar assay was combined with clinically validated biochemical markers of ECM synthesis to evaluate ECM synthesis over time. To validate the model as a drug screening tool for novel anti-fibrotic compounds, two approved compounds for IPF, nintedanib and pirfenidone, and a compound in development, omipalisib, were tested.

**Methods:**

Primary human lung fibroblasts from healthy donors were cultured for 12 days in the presence of ficoll and were stimulated with TGF-β1 with or without treatment with an ALK5/TGF-β1 receptor kinase inhibitor (ALK5i), nintedanib, pirfenidone or the mTOR/PI3K inhibitor omipalisib (GSK2126458). Biomarkers of ECM synthesis were evaluated over time in cell supernatants using ELISAs to assess type I, III, IV, V and VI collagen formation (PRO-C1, PRO-C3, PRO-C4, PRO-C5, PRO-C6), fibronectin (FBN-C) deposition and α-smooth muscle actin (α-SMA) expression.

**Results:**

TGF-β1 induced synthesis of PRO-C1, PRO-C6 and FBN-C as compared with unstimulated fibroblasts at all timepoints, while PRO-C3 and α-SMA levels were not elevated until day 8. Elevated biomarkers were reduced by suppressing TGF-β1 signalling with ALK5i. Nintedanib and omipalisib were able to reduce all biomarkers induced by TGF-β1 in a concentration dependent manner, while pirfenidone had no effect on α-SMA.

**Conclusions:**

TGF-β1 stimulated synthesis of type I, III and VI collagen, fibronectin and α-SMA but not type IV or V collagen. Synthesis was increased over time, although temporal profiles differed, and was modulated pharmacologically by ALK5i, nintedanib, pirfenidone and omipalisib. This prolonged 12-day Scar-in-a-Jar assay utilising biochemical markers of ECM synthesis provides a useful screening tool for novel anti-fibrotic compounds.

## Background

Most drug candidates for pulmonary fibrosis fail in human clinical trials [1, 2]. To reduce the attrition rates in the clinic it is essential that novel anti-fibrotic compounds are screened in reliable and disease relevant pre-clinical models of fibroproliferative diseases. It is important that these preclinical models replicate key events in human pulmonary fibrosis such as dysregulated fibroblast activity and aberrant remodeling of the extracellular matrix (ECM) [3].

Pulmonary fibrosis includes several lung disorders characterized by the formation of excessive scar tissue in the lungs. Idiopathic pulmonary fibrosis (IPF) is a particularly severe and progressive form [4], with a mean survival of 3–5 years after the time of diagnosis [5]. The incidence of IPF in Europe and North America has risen in recent years and is estimated to range between 2.8 and 18 cases per 100.000 people per year [6, 7]. During the development of IPF, healthy tissue is replaced by rigid ECM, destroying the lung architecture and leading to disrupted gas exchange and ultimately respiratory failure and death [3]. Transforming growth factor (TGF)-β1 plays a critical role in the differentiation of fibroblasts into myofibroblasts, which in turn produce ECM proteins driving the abnormal repair response and scar formation in IPF [8, 9]. During the progression of fibrosis, ECM alignment and composition is altered [10]. Imbalanced ECM remodelling leads to increased release of tissue- and pathology-specific protein fragments into the circulation [11, 12]. Such protease-generated fragments represent neo-epitopes which can be recognized by specific antibodies employed in enzyme-linked immunosorbent assays (ELISAs) and utilised as biomarkers. Some of these biomarkers have previously been shown to correlate with the progression of IPF [13]. Currently, no circulating biomarkers are routinely used for IPF in the clinic, neither for diagnosis, prognosis, prediction or monitoring. Some of the most commonly studied biomarkers include SP-D and KL-6, reflecting epithelial injury; MMP-7, periostin and ECM neo-epitopes such as C1M, C3M, C6M and CRPM reflecting ECM remodelling [14–16].

The first effective disease-modifying drugs to be approved by the U.S. Food and Drug Administration (FDA) and European Medicines Agency (EMA) were pirfenidone and nintedanib, which have succeeded in attenuating lung function decline in patients with IPF [17, 18]. There is still no cure for IPF, thus new therapeutic options are being explored [19, 20]. One group of therapies that is being tested in clinical trials is inhibitors of the mammalian target of rapamycin (mTOR). These were initially introduced into clinical practice to prevent transplant rejection and later to treat mTOR diseases such as lymphangioleiomyomatosis [21, 22]. The anti-fibrotic effect of the mTOR inhibitors is mediated by a decrease of type I and III collagen synthesis [23]. Omipalisib (GSK2126458), a potent inhibitor of mTOR and phosphatidylinositol 3-kinase (PI3K) [24], has been tested in a randomised, placebo-controlled study of IPF, which found that omipalisib has an acceptable tolerability and that target engagement was confirmed (NCT01725139) [25]. Additionally, recent data from a proof of mechanism study indicate that omipalisib reduces collagen synthesis as shown by a rapid decrease of the biomarkers PRO-C3 and PRO-C6 in serum of IPF patients [26]. By using human lung fibroblasts, Woodcock et al. showed that TGF-β1 signalling through mTOR is critical for fibrogenesis, by demonstrating that mTOR signalling, was required for type I collagen deposition in vitro [27]. Additional results from precision-cut IPF lung slices confirmed that synthesis of type I collagen, as measured by PRO-C1 ELISA in the supernatants, was mediated through mTOR [27].

Complex in vitro models of fibrogenesis using primary human lung fibroblasts offer a physiologically relevant alternative to in vivo models which may not fully recapitulate the complex pathogenesis exhibited by IPF patients [28, 29]. Cells grown in a crowded, pseudo-3D environment more closely resemble the physiological environment and the pathological ECM compared to standard 2D cell cultures [30]. The Scar-in-a-Jar model developed by Chen et al. [31] uses healthy human lung fibroblasts grown in medium with macromolecules (ficoll or dextran sulphate) to provide crowding conditions. Ficoll contains neutral macromolecules of 70 and 400 kDa that occupy the space in the well, making it unavailable for other molecules and thereby causing the excluded volume effect [32]. These crowded conditions cause increased interactions between the substrate and their respective enzymes, increasing enzymatic activity and accelerating collagen deposition [33]. The use of ficoll allows us to mimic the dense cellular environment within the ECM and improve the release of collagen pro-peptides as compared to systems without crowded conditions. Furthermore, Chen et al. demonstrated that the presence of ficoll leads to a higher degree of cross-linking in the deposited collagen [31], thus better representing in vivo conditions. Ascorbic acid is another crucial component in this model, as it is essential for the post-translational modification of proline to hydroxyproline that allows the correct folding of collagens during synthesis. The addition of human TGF-β1 induces fibroblast activation and differentiation to myofibroblasts, increasing ECM production and thereby imitating fibrogenesis [31]. Chen et al. showed that within 6 days the collagen deposition doubled in the Scar-in-a-Jar assay as compared with non-crowded cultures and that the addition of TGF-β1 increased the deposition 2-fold as compared with a crowded culture without stimulation. Furthermore, they showed a more aligned pattern of the fibrillar type I collagen and an effect of potential anti-fibrotic compounds on type I collagen deposition. Interestingly, the addition of a crowding agent induced complete cleavage of the type I collagen C-propeptide, whereas the fibroblasts in the non-crowded culture conditions continued to secrete intact procollagen. Recently, Good et al. described a 72 h high content screening Scar-in-a-Jar assay utilizing fibroblasts from IPF patients, that allowed the quantification of disease-relevant ECM deposition in vitro [34].

**Fig. 1:**
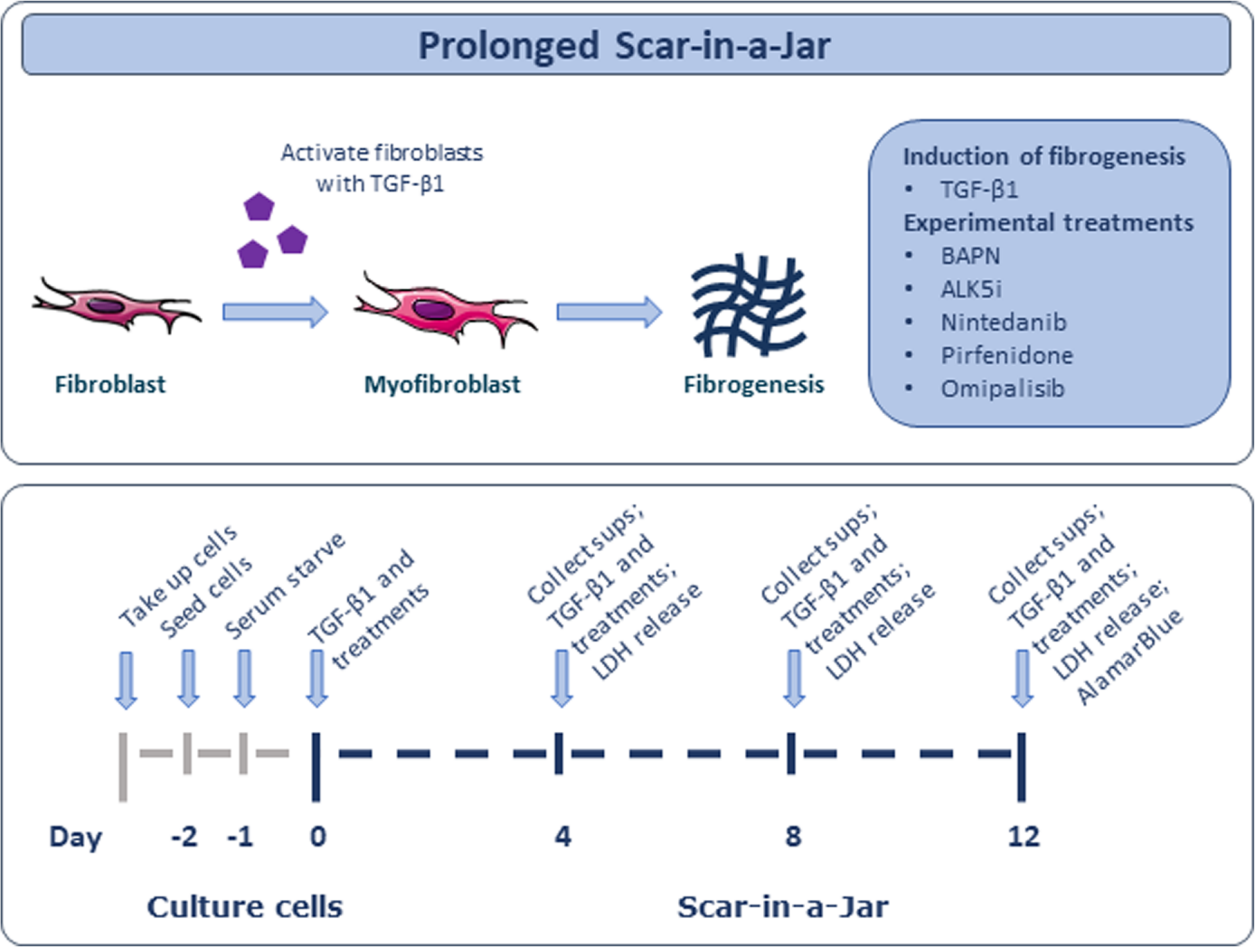
Prolonged Scar-in-a-Jar as a model of fibrogenesis. Primary lung fibroblasts from healthy donors were cultured in the presence of ficoll to promote a crowded environment. Fibroblasts were stimulated with TGF-β1 and differentiated into myofibroblasts that produced high amounts of extracellular matrix proteins. Cells were cultured for 12 days with medium changes at day 4 and 8

Here, a prolonged Scar-in-a-Jar model was used to allow for correct collagen processing, and biochemical markers quantified ECM synthesis for up to 12 days. These markers include PRO-C3 and PRO-C6, biomarkers of type III and VI collagen synthesis, respectively, which have been found to be increased in blood from progressing IPF patients as compared to stable IPF patients in the PROFILE (Prospective Observation of Fibrosis in the Lung Clinical Endpoints) study [35]. In addition, serum levels of a marker of myofibroblasts, alpha-smooth muscle actin (α-SMA), has also been shown to be increased in IPF patients compared to healthy controls [36]. The effect of TGF-β1 stimulation on ECM synthesis was investigated after 4, 8, and 12 days to determine the kinetics of the different ECM proteins produced. Further, the use of the prolonged Scar-in-a-Jar model as a drug screening tool for novel anti-fibrotic compounds was evaluated by testing nintedanib, pirfenidone and the mTOR/PI3K inhibitor omipalisib. The model is depicted in Fig. 1.

## Methods

### Fibroblast cell culture

Primary human lung fibroblasts in passage 5 and 9 were purchased from Lonza (Basel, Switzerland) (cat. no. CC-2512) and kindly provided by GSK (Stevenage, UK). All patients gave full consent and procedures were performed in line with research ethics committee approval. Human biological samples were sourced ethically, and their research use was in accord with the terms of the informed consents under an IRB/EC approved protocol. Cells were grown to confluence and seeded at a density of 30,000 cells/well in 48-well plates in high serum medium (10% fetal bovine serum (FBS) (cat. no. F7524, Sigma-Aldrich, St. Louise, Missouri, USA) in Dulbecco’s modified eagle medium (DMEM) + GlutaMax (cat. no. 31966, Gibco, Life Technologies, Carlsbad, California, USA)) at day -2. The cells were starved in low serum medium (0.4% FBS DMEM) at day -1 to avoid interference with biomarker measurements. At day 0, 200 μL of 1 ng/mL TGF-β1 (cat. no. 100-B-010/CF, R&D system, Minneapolis, Minnesota, USA) diluted in low serum medium containing ficoll (56.25 mg/mL ficoll 70, 37.5 mg/mL ficoll 400; cat. no. F2878, Sigma-Aldrich) and 1.5% L-ascorbic acid, phosphate magnesium salt, n-hydrate (cat. no. 013–19,641, Wako, Osaka, Japan) were added to each well. 100 μL of the treatments were added as appropriate: beta-aminopropionitrile (BAPN) (cat. no. A3134, Sigma-Aldrich) in 0.2 mM; activin receptor-like kinase 5 (ALK5)/type I TGF-β receptor kinase inhibitor (ALK5i) (SB-525334, cat. no. S8822, Sigma-Aldrich) and nintedanib (cat. no. 656247–17-5, Kemprotec Ltd., Smailthorn, Carnforth, UK) in 1 nM, 10 nM, 100 nM, 1 μM and 10 μM; pirfenidone (kindly provided by Grünenthal, Aachen, Germany) in 30, 100, 300, 1000 and 3000 μM; and omipalisib (GSK2126458, cat. no. CT-GSK458, Chemietek, Indianapolis, Indiana, USA) in 0.01, 0.1, 1, 10 and 100 nM. ALK5i, nintedanib and omipalisib were diluted in dimethyl sulfoxide (DMSO) and concentration was kept constant at 0.1%. The corresponding vehicle consisted of 0.1% DMSO. For pirfenidone, due to solubility issues DMSO content was dependent on pirfenidone concentration and the following concentrations of DMSO were included as vehicles: 0.03, 0.1, 0.3, 1 and 3%. Cells were incubated at 37 °C with 95% O_2_ and 5% CO_2_ and cultured for 12 days. TGF-β1 medium and treatments were prepared freshly and exchanged at day 0, 4 and 8. Supernatants from day 4, 8 and 12 were stored at -20 °C until biomarker assessments by ELISA. Three replicates with four technical replicates testing nintedanib, pirfenidone, ALK5i and omipalisib were performed using the primary fibroblasts from Lonza. The experiment testing BAPN was performed once with four technical replicates using the primary fibroblasts from GSK.

### Matrix cleavage

At day 12, the matrix deposited at the bottom of the wells together with the cells was washed twice with phosphate-buffered saline (PBS) and stored at -20 °C. Before digestion, the matrix was thawed to room temperature and washed gently with digestion buffer (50 mM TRIS, 36 mM CaCl_2_, pH 7.5). 200 μL of collagenase (cat. no. C9891, Sigma-Aldrich) diluted in digestion buffer (130 μg/mL) was added to each well and incubated at 37 °C for 24 h. The reaction was stopped by adding 20 μL of stop solution (1 tablet of cOmplete Mini Protease Inhibitor Cocktail (cat. no. 11836153001, Roche, Basel, Switzerland) dissolved in 2.5 ml of Milli-Q water).

### Metabolic activity assessed by Alamar blue

To assess the effects of the inhibitors on cell health, Alamar Blue (cat. no. DAL1100, Invitrogen, Carlsbad, California, USA) was used to quantify cellular metabolism at day 0 and 12. In brief, Alamar Blue was diluted 1:10 in low serum medium and 200 μL was added to each well and incubated for 2 h at 37 °C in 5% CO_2_. In the presence of metabolically active cells, resazurin is reduced to fluorescent resorufin, which can be quantified by a colorimetric change and fluorescent signal. After incubation, 160 μL was transferred to black 96-well plates and the fluorescence was measured using 540 nm as excitation wavelength and subtracting the background measured using 590 nm as emission wavelength on an ELISA reader.

### Cytotoxicity assessed by LDH release

To assess the effects of compound incubation on cytotoxicity, release of lactate dehydrogenase (LDH) from damaged cells was assessed at day 4, 8 and 12 using the Cytotoxicity Detection Kit^PLUS^ (LDH) (cat. no. 04744934001, Roche) according to the manufacturer’s instructions. In brief, 50 μL of cell supernatant was mixed with 50 μL of reaction mixture in uncoated, clear, flat bottomed 96-well plates. Plates were incubated for 30 min at 20 °C in the dark, shaking 300 rpm, followed by 25 μL stop solution. Absorbance was measured at 492 nm with 690 nm as a reference on an ELISA reader, subtracting the background. To determine the maximum LDH release possible, one well with cells treated with TGF-β1 was lysed at day 4, 8 and 12, using 200 μL of lysis solution diluted 1:20 in low serum medium and allowed to incubate for 15 min at 37 °C in 5% CO_2_ (lysis control).

### Biomarker measurements

Collagen synthesis, fibronectin and α-SMA were evaluated in cell supernatants collected at day 4, 8, and 12 using specific competitive ELISAs (Nordic Bioscience, Herlev, Denmark). The PRO-C1 assay (cat. no. 2800) measures the N-terminal pro-peptide of type I collagen, describing the formation of type I collagen [37]. The PRO-C3 assay (cat. no. 1700) measures the neo-epitope of the N-terminal pro-peptide of type III collagen after cleavage from the pro-collagen molecule and describes the formation of type III collagen [38]. The PRO-C4 assay (cat. no. 8000) measures an internal epitope in the 7S domain of type IV collagen [39]. The PRO-C5 assay (cat. no. 3000) measures the neo-epitope of the C-terminal pro-peptide of type V collagen after cleavage from the pro-collagen molecule and describes the formation of type V collagen [40, 41]. The PRO-C6 assay (cat. no. 4000) measures the C-terminal of the released C5 domain of the type VI collagen α3 chain, also referred to as endotrophin, and describes the formation of type VI collagen [42]. The FBN-C assay (cat. no. N101–00) measures the C-terminal of fibronectin [43] and the α-SMA assay (cat. no. N132–00) measures the acetylated N-terminal of α-SMA [36]. The detailed protocols for each assay can be found in the specific references. Furthermore, αCTX-1 (Alpha CrossLaps® (CTX-I) ELISA, cat. no. AC-04F1, Immunodiagnostic System Nordic A/S, Copenhagen, Denmark), a marker of type I collagen cross-linking, was assessed in the cleaved matrix at day 12.

### Staining

Primary human lung fibroblasts (cat. no. CC-2512, Lonza) were grown to confluence and seeded at a density of 22,000 cells/well in 8-well EZ glass slides (cat. no. PEZGS0816, Millipore, Massachusetts, USA) and cultivated for 12 days following the procedure described in “fibroblast cell culture”. Fibroblasts were unstimulated (control) or stimulated with TGF-β1. The wells were washed three times with PBS followed by fixation with 4% formaldehyde (Sigma-Aldrich) for 2 h. The fixed cells were washed three times with PBS before adding 300 μL peroxidase for 10 min. The wells were washed once with PBS and blocked with 300 μL 2% skim milk diluted in PBS for 15 min before adding 300 μL of monoclonal mouse antibody targeting type I and III collagen (cat. no. Ab6308 and Ab6310, Abcam, Cambridge, UK) or α-SMA (cat. no. Ab7817, Abcam) diluted 1:100 in 2% skim milk and incubated overnight. The wells were washed three times with PBS and incubated with 300 μL of HRP-labelled goat anti-mouse antibody (cat. no. K4001, Dako, Glostrup, Denmark) for 30 min. Wells were washed 3 times with PBS before adding 300 μL of substrate (1 drop of chromogen diluted in 1 mL substrate buffer; cat. no. K3468, Dako) and incubated for 1–15 min until color change was evident. Wells were washed 3 times with PBS and counterstained with Mayer’s hematoxylin (1.5 g hematoxylin, 0.2 g sodium iodate, 50 g potassium aluminum sulfate, 1 g citric acid monohydrate and 1 L MilliQ water) for 12 s and rinsed with tap-water before allowing to dry. All the steps were performed at room temperature. Pictures of ×10 magnification were obtained using an Olympus DP71 digital camera connected to an Olympus BX60 microscope.

### Statistical analysis

Statistical significance was determined using t-test or two-way ANOVA with Sidak’s or Dunnett’s multiple comparisons test. Data are plotted as raw values or percentage of the TGF-β1 control or lysis control. Data are presented as mean ± SD of 3 separate experiments each with 4 replicates/treatment. Treatment data are plotted as line graphs comparing biomarker data from day 4, 8 and 12 or vehicle and treatment. All statistical tests were performed in GraphPad Prism software v.6 (GraphPad Software, San Diego, CA). *P*-values lower than 0.05 were considered significant.

## Results

### The fibrogenic potential of lung fibroblasts stimulated with TGF-β1

**Fig. 2:**
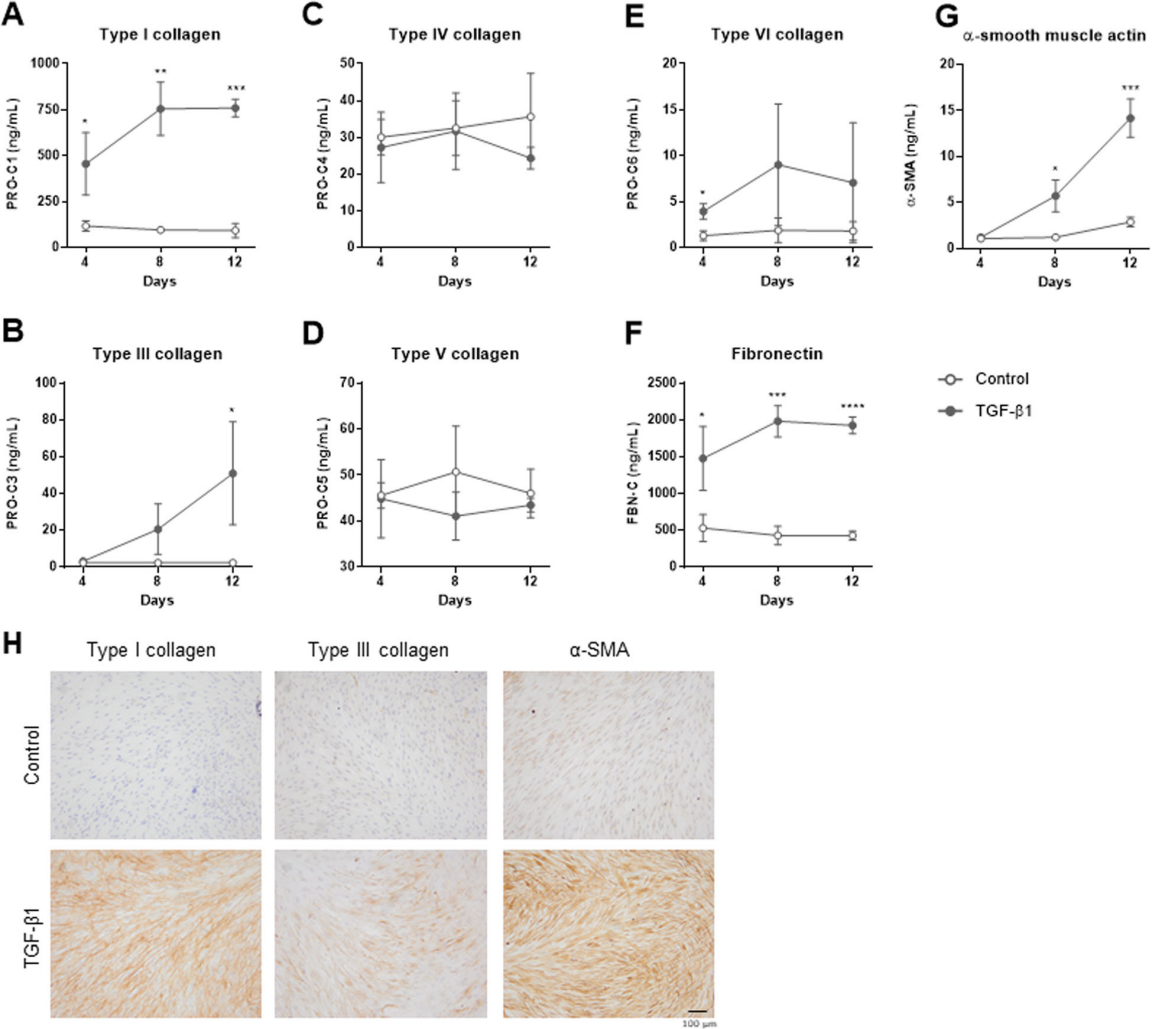
TGF-β1 stimulated synthesis of type I, III and VI collagen, fibronectin and α-SMA but not type IV and V collagen. Biomarkers of extracellular matrix synthesis and fibroblast activation released from lung fibroblast cultured with TGF-β1 or without TGF-β1 (control) are shown at day 4, 8 and 12. Synthesis of type I (**a**), III (**b**), IV (**c**), V (**d**) and VI (**e**) collagens are measured by PRO-C1, PRO-C3, PRO-C4, PRO-C5 and PRO-C6, respectively. FBN-C measures production of fibronectin (**f**) and α-smooth muscle actin (α-SMA) is a surrogate marker for activated fibroblasts (myofibroblasts)(**g**). Data are shown as mean ± SD of 3 separate experiments, each with 4 replicates/treatment. Data are analyzed by student’s t-test. *P < 0.01; **P < 0.01; ***P < 0.001; ****p < 0.0001. (**h**) Staining of type I and III collagen and α-SMA in wells with lung fibroblasts at day 12. Fibroblasts were unstimulated (control) or stimulated with TGF-β1. Pictures are ×10 magnification

The fibrogenic potential of lung fibroblasts was evaluated by assessing ECM production in response to TGF-β1 by assessing biomarkers of type I (PRO-C1), III (PRO-C3), IV (PRO-C4), V (PRO-C5) and VI (PRO-C6) collagen synthesis as well as fibronectin (FBN-C) in cell supernatants. Additionally, the expression of α-SMA, a marker of the activated myofibroblast, was assessed. After 4 days of culture, fibroblasts stimulated with TGF-β1 produced significantly higher levels of type I collagen (PRO-C1; 4-fold, *P* < 0.05; Fig. 2a), type VI collagen (PRO-C6; 3-fold, P < 0.05; Fig. 2e) and fibronectin (FBN-C; 3-fold, P < 0.05; Fig. 2f) as compared to unstimulated cells, and peaked at day 8 with 8- (*P* = 0.001), 5- (*P* = 0.1) and 5-fold (*P* = 0.0004) increases, respectively, and remained elevated until day 12. Production of type III collagen (PRO-C3; Fig. 2b) and α-SMA (Fig. 2g) were at the level of unstimulated cells at day 4 and increased 9- (*P* = 0.08) and 4-fold (*P* = 0.01) at day 8 and additionally to 23- (*P* = 0.04) and 5-fold (*P* = 0.0008), respectively, at day 12. Type IV (PRO-C4) and V (PRO-C5) collagen production was not affected by TGF-β1 stimulation, and levels remained constant throughout the experiment (Fig. 2c-d).

To further characterize the model, we stained for α-SMA as a marker of fibroblast activation and the two most prominent collagens, type I and III, to visualize the ECM deposition at day 12 (Fig. 2h). Type I collagen fibrils were clearly visible in the ECM surrounding fibroblasts stimulated with TGF-β1, whereas wells with unstimulated fibroblasts contained small amounts of type I collagen staining. TGF-β1 stimulation also induced type III collagen synthesis, albeit to a lesser degree. Staining of α-SMA showed a slight activation of unstimulated cells, possibly caused by them growing on plastic, but TGF-β1 stimulation increased the signal dramatically.

### Collagen cross-linking occurs in the prolonged Scar-in-a-Jar model and can be modulated

**Fig. 3:**
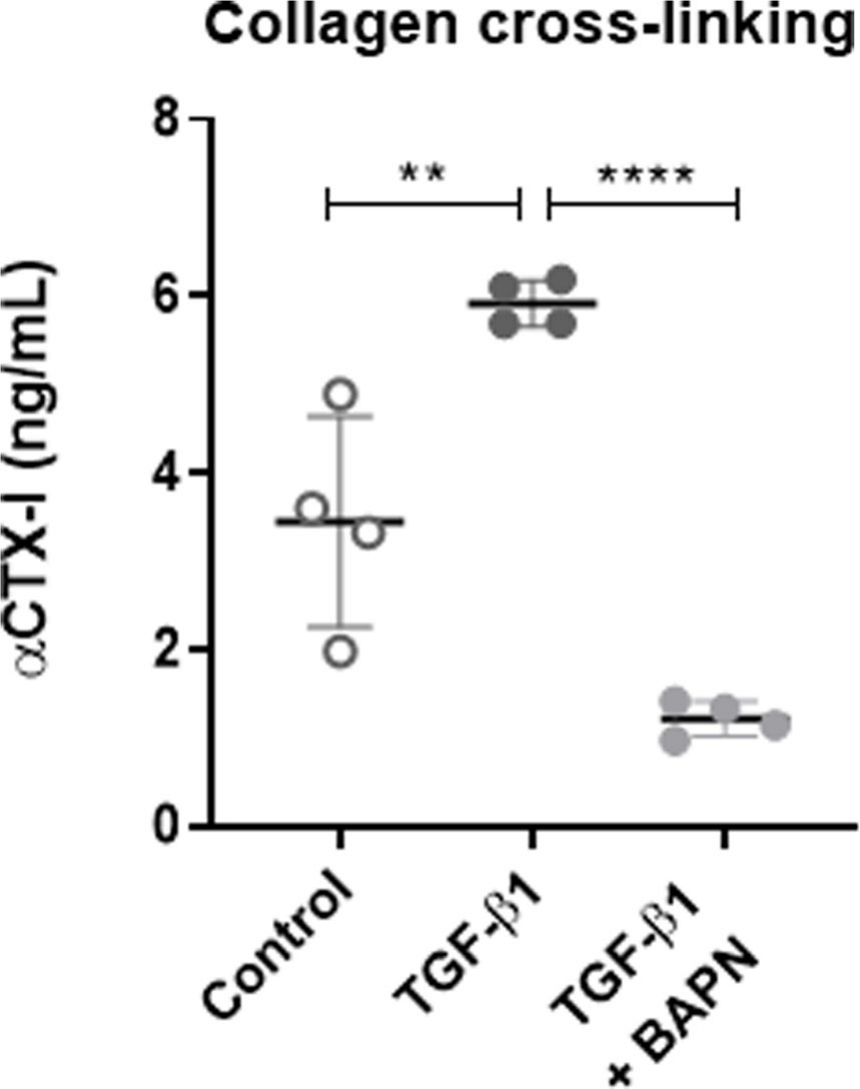
TGF-β1 induced collagen cross-linking which could be modulated by a LOX inhibitor. Levels of type I collagen cross-linking (αCTX-I) were assessed in collagenase-cleaved matrix produced by lung fibroblasts on day 12. Fibroblasts were cultured without stimulation (control), with TGF-β1 alone or with TGF- β1 and the pan-lysyl oxidase (LOX) inhibitor beta-aminopropionitrile (BAPN). Data are shown as mean ± SD of 4 replicates and analyzed by student’s t-test comparing TGF- β1 to control or TGF-β1 + BAPN. **P < 0.01; ****P < 0.0001

Collagen cross-linking is a key feature of fibrogenesis. To determine whether collagen cross-linking occurs in this assay fibroblasts were stimulated with TGF-β1 in the presence or absence of the pan-lysyl oxidase (LOX) inhibitor BAPN. Cross-linking was evaluated by assessing αCTX-I, a marker of cross-linking in the C-terminal telopeptide of type I collagen, in collagenase-cleaved matrix (Fig. 3). Matrix originating from fibroblasts stimulated with TGF-β1 had significantly higher levels of αCTX-I as compared with matrix from unstimulated fibroblasts (*P* = 0.007), indicating that collagen cross-linking does occur in the prolonged Scar-in-a-Jar model. In addition, TGF-β1 stimulation combined with BAPN treatment significantly reduced αCTX-I levels by 6-fold compared to untreated fibroblasts stimulated with TGF-β1 only (*p* < 0.0001), indicating that cross-linking may be modified in this model.

### The fibrogenic response can be modulated by inhibiting TGF-β1 signaling

**Fig. 4:**
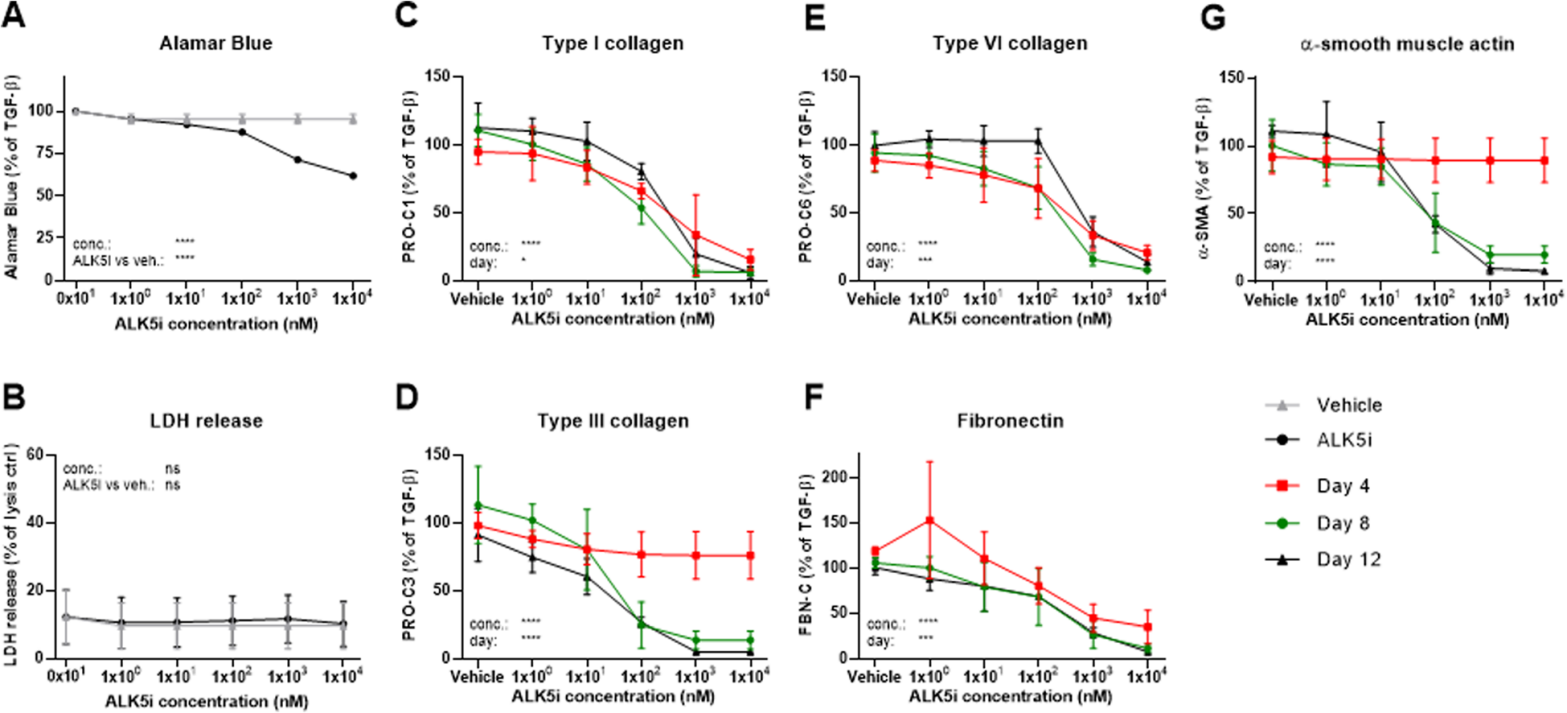
Inhibition of TGF-β1 signaling by an ALK5 inhibitor reduced fibrogenesis. Lung fibroblasts were stimulated with TGF-β1 and treated with 1 × 10^0^, 1 × 10^1^, 1 × 10^2^, 1 × 10^3^ or 1 × 10^4^ nM activin receptor-like kinase 5 (ALK5)/type I TGF-β receptor kinase inhibitor (ALK5i) or vehicle (0.1% DMSO). **a** Metabolic activity was assessed by Alamar Blue at day 12 and data are presented as percentages of the TGF-β1 control for vehicle and ALK5i. **b** Cytotoxicity was assessed by lactate dehydrogenase (LDH) release at day 4 and data are presented as percentage of the maximum LDH release determined by cell lysis for vehicle and ALK5i. **c**-**g** Biomarkers of ECM synthesis (type I (PRO-C1), III (PRO-C3) and VI (PRO-C6) collagen and fibronectin (FBN-C)) and fibroblast activation (α-SMA) were measured in the supernatant at day 4, 8 and 12. Data are presented as percentage of the TGF-β1 control for day 4, 8, and 12. All data are shown as mean ± SD of 3 separate experiments each with 4 replicates/treatment and analyzed by two-way ANOVA with Sidak’s or Dunnett’s multiple comparisons test comparing ALK5i to vehicle. *P < 0.05; **P < 0.01; ***P < 0.001; ****P < 0.0001

To investigate whether fibrogenesis could be modulated in this system, the TGF-β1 receptor kinase inhibitor ALK5i, known to inhibit collagen production, was used at five different concentrations on TGF-β1-stimulated fibroblasts as a positive control and its effects on ECM synthesis was evaluated. ALK5i had a concentration-dependent effect on the metabolic activity measured by Alamar Blue, with significant decreases after treatment with 100 nM (88%, *P* = 0.0084), 1 μM (71%, *P* < 0.0001) and 10 μM (62%, P < 0.0001) ALK5i as compared with vehicle (95%; Fig. 4a). However, a minimal LDH release (*P* = 0.7) indicated that the cytotoxic effect of ALK5i was negligible (Fig. 4b). ALK5i concentration-dependently and significantly lowered the levels of PRO-C1, PRO-C6 and FBN-C at all days (Fig. 4c, e, f). An effect on PRO-C3 and α-SMA was seen on day 8 and 12, but not on day 4 where production was not initiated (Fig. 4d, g).

### Effect of anti-fibrotic compounds on ECM synthesis in the prolonged Scar-in-a-Jar model

To further evaluate the prolonged Scar-in-a-Jar model, the potency of anti-fibrotic compounds in the clinic or in development were tested. Fibroblasts stimulated with TGF-β1 were treated with nintedanib, pirfenidone, or omipalisib and the ECM biomarkers that were found to respond to TGF-β1 stimulation were assessed in supernatant collected at day 4, 8 and 12 to evaluate the anti-fibrotic effects on fibroblasts.

**Fig. 5:**
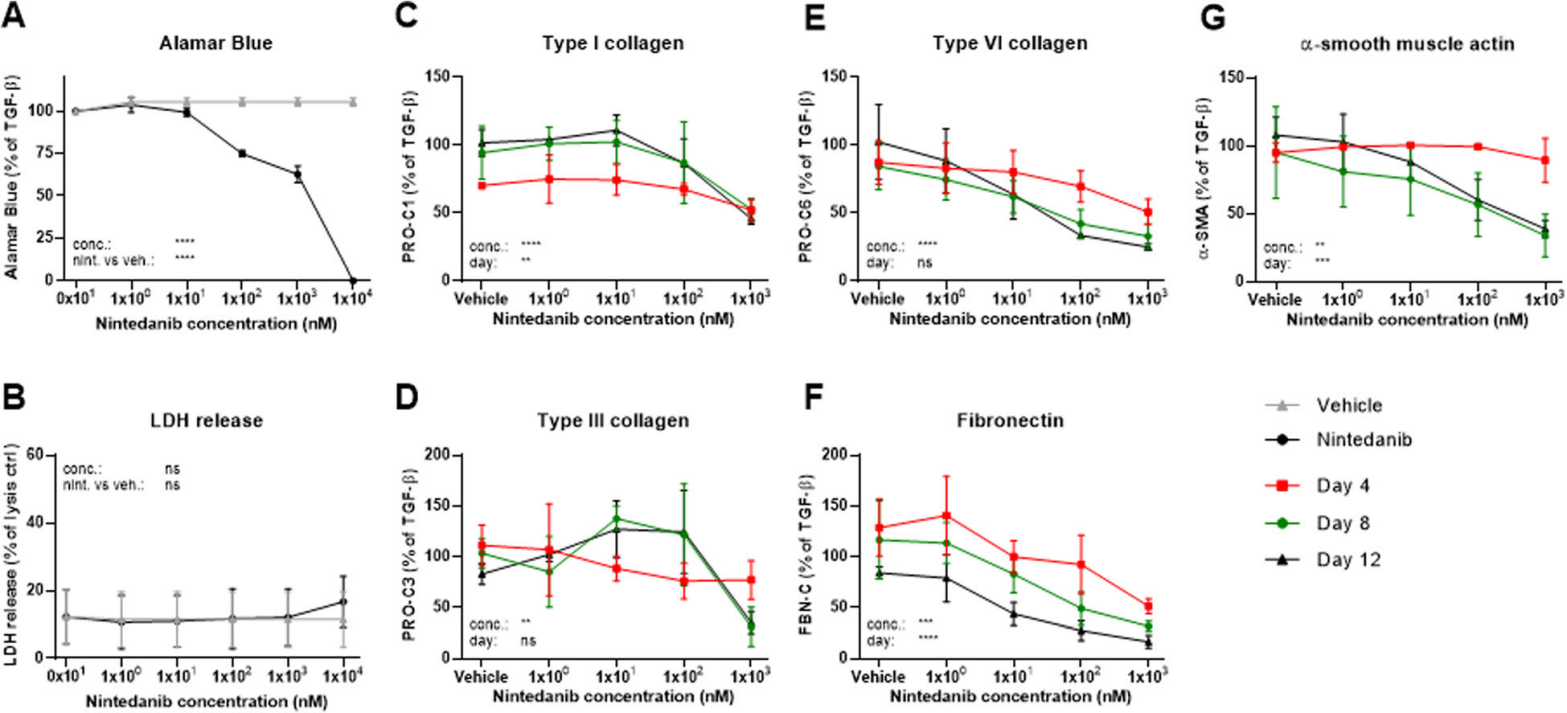
Nintedanib reduced fibrogenesis and effects were more evident at later timepoints. Lung fibroblasts were stimulated with TGF-β1 and treated with 1 × 10^0^, 1 × 10^1^, 1 × 10^2^, 1 × 10^3^ or 1 × 10^4^ nM nintedanib or vehicle (0.1% DMSO). **a** Metabolic activity was assessed by Alamar Blue at day 12 and data are presented as percentages of the TGF-β1 control for vehicle and nintedanib. **b** Cytotoxicity was assessed by lactate dehydrogenase (LDH) release at day 4 and data are presented as percentage of the maximum LDH release determined by cell lysis for vehicle and nintedanib. **c**-**g** Biomarkers of ECM synthesis (type I (PRO-C1), III (PRO-C3) and VI (PRO-C6) collagen and fibronectin (FBN-C)) and fibroblast activation (α-SMA) were measured in the supernatant at day 4, 8 and 12. Data are not shown for the highest concentration of nintedanib which significantly reduced Alamar Blue. Data are presented as percentage of the TGF-β1 control for day 4, 8, and 12. All data are shown as mean ± SD of 3 separate experiments each with 4 replicates/treatment and analyzed by two-way ANOVA with Sidak’s or Dunnett’s multiple comparisons test comparing nintedanib to vehicle. ns non-significant; *P < 0.05; **P < 0.01; ***P < 0.001; ****P < 0.0001

Nintedanib significantly decreased metabolic activity, as determined by Alamar Blue, as compared to vehicle at concentrations of 100 nM, 1 μM and 10 μM to 75, 63 and 0%, respectively (all *P* < 0.0001), of the TGF-β1 control (Fig. 5a). LDH release was not significantly changed in response to nintedanib, however at the highest concentration (10 μM) showed a small trend for elevated LDH release (Fig. 5b). Based on the 100% reduction in Alamar Blue for the highest concentration of nintedanib, we omitted this concentration in the following biomarker analyses. Nintedanib concentration-dependently decreased PRO-C1, PRO-C6 and FBN-C at all days with largest effects observed at day 12 where levels decreased to 46, 25 and 16% of the TGF-β1 control as compared with the respective vehicle controls (102, 102 and 84%) (Fig. 5c, e, f). PRO-C3 and α-SMA levels were not altered at day 4 where production of these had not initiated, but were decreased in response to nintedanib at day 8 and 12 to 31% for PRO-C3 and 34% for α-SMA of the TGF-β1 control at day 8 as compared with the vehicle controls (103 and 95%) (Fig. 5d, g).

**Fig. 6:**
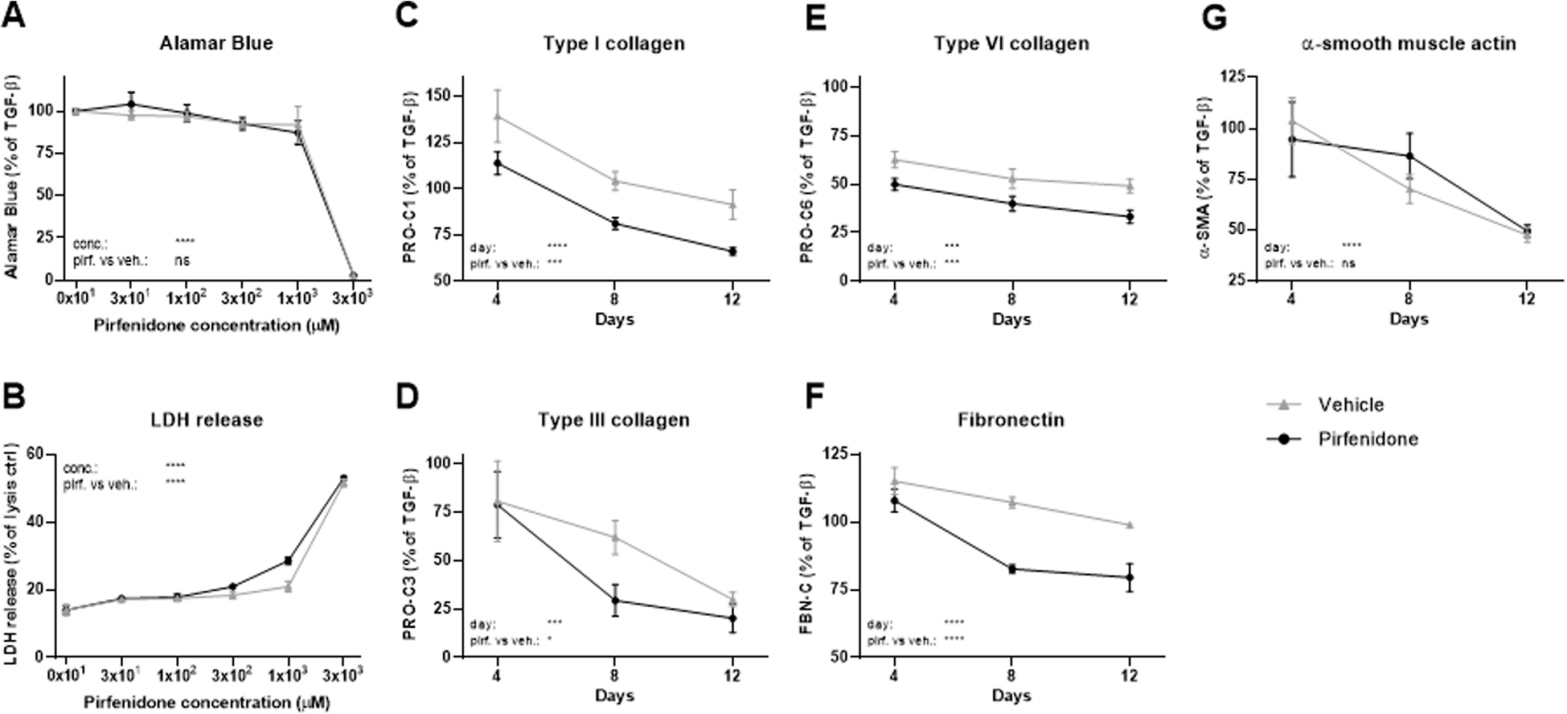
Pirfenidone reduced matrix protein synthesis but had no effect on fibroblast activation. Lung fibroblasts were stimulated with TGF-β1 and treated with 3 × 10^1^, 1 × 10^2^, 3 × 10^2^, 1 × 10^3^ or 3 × 10^3^ μM pirfenidone or corresponding vehicle (0.03, 0.1, 0.3, 1% or 3% DMSO). **a** Metabolic activity was assessed by Alamar Blue at day 12 and data are presented as percentages of the TGF-β1 control for vehicle and pirfenidone. **b** Cytotoxicity was assessed by lactate dehydrogenase (LDH) release at day 4 and data are presented as percentage of the maximum LDH release determined by cell lysis for vehicle and pirfenidone. **c**-**g** Biomarkers of ECM synthesis (type I (PRO-C1), III (PRO-C3) and VI (PRO-C6) collagen and fibronectin (FBN-C)) and fibroblast activation (α-SMA) were measured in the supernatant at day 4, 8 and 12. Data are shown for 1 × 10^3^ μM pirfenidone and its corresponding vehicle and are presented as percentage of the TGF-β1 control over time. All data are shown as mean ± SD of 3 separate experiments each with 4 replicates/treatment and analyzed by two-way ANOVA with Sidak’s multiple comparisons test comparing pirfenidone to vehicle at day 4, 8 and 12. ns non-significant; *P < 0.05; **P < 0.01; ***P < 0.001; ****P < 0.0001

Pirfenidone at the highest concentration used (3000 μM) and its corresponding vehicle (3% DMSO) both decreased Alamar Blue to 3% of the TGF-β1 control (*P* < 0.0001; Fig. 6a), and there was no difference between pirfenidone and its vehicle at any concentrations. LDH release was significantly increased in response to pirfenidone treatment at concentrations above 300 μM (21% vs. 18%, *P* = 0.004). At 3000 μM, LDH release increased to 53 and 52% of the lysis control for pirfenidone and its vehicle control, respectively (Fig. 6b). These results indicate that the solvent (increasing concentrations of DMSO) had a significant toxic effect on the cells which almost completely abolish metabolic activity at the highest concentration (3%). However, the cytotoxic effect was slightly but significantly higher for pirfenidone as compared to vehicle (0.3 and 1% DMSO) at concentrations of 300 and 1000 μM, indicating a mild cytotoxic effect of pirfenidone. Thus, based on the 97% reduction in metabolic activity and a significant increase in LDH release, the highest concentration of pirfenidone was omitted in the following biomarker analyses. Here, data are presented which compare one concentration of pirfenidone (1000 μM) to vehicle (1% DMSO) over time. No effects were seen for concentrations of pirfenidone below 300 μM. Graphs showing all concentrations of pirfenidone are included in Supplementary Figure 1. At day 4, no difference between pirfenidone and vehicle was observed for PRO-C1, PRO-C3, FBN-C or α-SMA (Fig. 6c, d, f, g). Only PRO-C6 showed a significant difference (*P* < 0.01), with decreases of PRO-C6 levels for 1000 μM pirfenidone of 50% as compared with 60% for vehicle (Fig. 6e). At day 8 and 12, pirfenidone significantly decreased levels of PRO-C1 (*P* = 0.002 and 0.02), PRO-C6 (*P* = 0.02 and 0.002) and FBN-C (*P* < 0.0001 and *P* = 0.01), while PRO-C3 was only significantly decreased at day 8 (*P* = 0.005) as compared to vehicle. Pirfenidone had no effect on α-SMA levels as compared to vehicle at any timepoint.

**Fig. 7:**
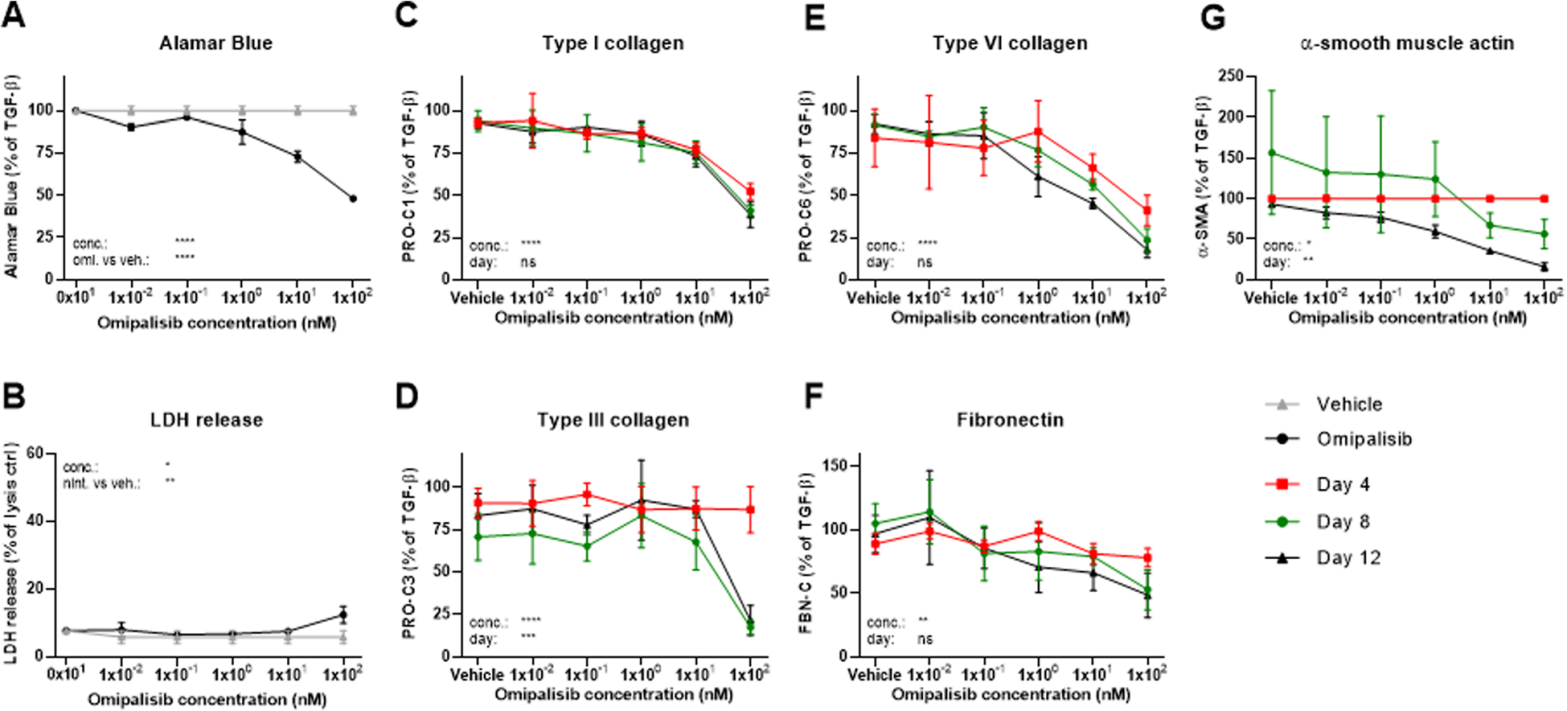
Omipalisib reduced fibrogenesis and effects were more evident at later timepoints. Lung fibroblasts were stimulated with TGF-β1 and treated with 1 × 10^− 2^, 1 × 10^− 1^, 1 × 10^0^, 1 × 10^1^ or 1 × 10^2^ nM omipalisib or vehicle (0.1% DMSO). **a** Metabolic activity was assessed by Alamar Blue at day 12 and data are presented as percentages of the TGF-β1 control for vehicle and omipalisib. **b** Cytotoxicity was assessed by lactate dehydrogenase (LDH) release at day 4 and data are presented as percentage of the maximum LDH release determined by cell lysis for vehicle and omipalisib. **c**-**g** Biomarkers of ECM synthesis (type I (PRO-C1), III (PRO-C3) and VI (PRO-C6) collagen and fibronectin (FBN-C)) and fibroblast activation (α-SMA) were measured in the supernatant at day 4, 8 and 12. Data are presented as percentage of the TGF-β1 control for day 4, 8, and 12. All data are shown as mean ± SD of 3 separate experiments each with 4 replicates/treatment and analyzed by two-way ANOVA with Sidak’s or Dunnett’s multiple comparisons test comparing omipalisib to vehicle. ns non-significant; *P < 0.05; **P < 0.01; ***P < 0.001; ****P < 0.0001

Omipalisib significantly decreased metabolic activity to 48% of the TGF-β1 control at the highest concentration (100 nM) as compared to 100% for the vehicle (P < 0.0001; Fig. 7a). Additionally, 100 nM omipalisib significantly increased LDH release to 12% of the lysis control as compared to 6% for the vehicle (*P* = 0.0003; Fig. 7b). Omipalisib concentration-dependently reduced all biomarker levels (Fig. 7c-g) with the largest effects observed for PRO-C3, PRO-C6 and α-SMA where levels were reduced to 22, 18 and 16%, respectively, of the TGF-β1 control at day 12 as compared with the respective vehicle controls (83, 92 and 94%). PRO-C3 and α-SMA levels were not altered at day 4 where production of these had not initiated, and for the other biomarkers, effects at day 4 were more modest than at later timepoints.

## Discussion

Many anti-fibrotic drug candidates fail when entering clinical trials, despite promising results obtained in a pre-clinical setting. This suggests that improved pre-clinical assays are needed to successfully translate the preclinical results to the clinical setting. Here, we propose a novel tool for drug screening that has the potential to aid the anti-fibrotic drug development process. We developed a prolonged Scar-in-a-Jar in vitro model and combined the assay with the measurement of clinically validated biomarkers of ECM synthesis in the cell supernatant. The model was validated by testing first a TGF-β1 receptor kinase inhibitor, and subsequently the two FDA approved anti-fibrotic compounds nintedanib and pirfenidone together with a mTOR/PI3K inhibitor in development, omipalisib. We found that stimulating primary lung fibroblasts with TGF-β1 increased the ECM synthesis and α-SMA expression over time, and that this was modulated by anti-fibrotic treatments in a concentration dependent manner.

Currently, many in vitro models only allow investigations of fibrogenesis in acute studies of 1–3 days [28, 34] although some models extend to a few weeks [44]. To our knowledge, this is the first time that a 12 days fibroblast culture has been combined with the investigation of ECM synthesis in the supernatant. The prolonged culture allows investigation of a drug in a system which is not limited to the acute response. In this study we investigated the ECM production after 4, 8 and 12 days of fibroblast culture, and observed that synthesis of different proteins occur at different times. Interestingly, type III collagen and α-SMA synthesis was not evident at day 4 but increased with TGF-β1 stimulation at day 8 and even more so at day 12. Levels of type I collagen and fibronectin peaked at day 8 and stayed elevated at day 12, while remaining biomarkers continued to increase over the whole course of the experiment. This could indicate that there is a continuous activation of fibroblasts and additive contribution to the ECM synthesis throughout the 12-day period. In the prolonged Scar-in-a-Jar model, TGF-β1 significantly induced accumulation of type I (PRO-C1), III (PRO-C3) and VI (PRO-C6) collagen, fibronectin (FBN-C) and α-SMA in comparison to unstimulated cells but did not stimulate type IV (PRO-C4) or V (PRO-C5) collagen production. Type I and III collagen are the main ECM proteins associated with fibrogenesis, and TGF-β1 has previously been identified as a stimulator of type I and III collagen production by lung fibroblasts in vitro [45]. In line with our data, Walker et al. observed a small increase in fibronectin expression in airway fibroblasts in response to TGF-β stimulation after one day of culture while the increase was more pronounced after 20 days [44]. This underlines the importance of the temporal aspect when investigating fibrotic processes. Normally, fibroblasts do not produce type IV collagen, but Roach et al. showed that myofibroblasts had increased type IV collagen mRNA expression upon stimulation with TGF-β1 [46]. Tuan et al. demonstrated that fibroblasts in a fibroplasia model were able to produce type V collagen, albeit to a much smaller extent than type I collagen [47]. Additionally, Knüppel et al. observed that TGF-β stimulation of primary fibroblasts resulted in increased type V collagen mRNA expression and protein concentration [48]. This indicates that the conditions in the prolonged Scar-in-a-Jar model are not favorable for the production of type IV and V collagen, or that amounts are too small to observe. Using a different fibrogenic stimuli, e.g. platelet-derived growth factor (PDGF), may induce fibrogenesis with a different ECM protein profile. Furthermore, the degree of collagen cross-linking in the prolonged Scar-in-a-Jar model was significantly elevated by TGF-β1 stimulation and could be abolished by a pan-LOX inhibitor. Hence, providing a model which could be used to investigate the effects of current anti-fibrotic compounds on collagen cross-linking, an effect which could promote fibrolysis and be beneficial for patients with pulmonary fibrosis.

ALK5i, an inhibitor of the type I TGF-β receptor kinase, was able to significantly decrease the TGF-β1-induced levels of PRO-C1, PRO-C3, PRO-C6, FBN-C, and α-SMA. Thus, the pro-fibrotic effects that TGF-β1 exerts on lung fibroblasts can be modulated in this system, and the combination of the prolonged Scar-in-a-Jar model and biochemical markers of ECM synthesis may be a useful tool for evaluating anti-fibrotic compounds. In line with this, Epstein et al. showed that an ALK5i was able to reduce α-SMA and type I collagen mRNA levels after 24 h in normal fibroblasts cultivated in an IPF conditioned matrix [49].

Nintedanib and omipalisib were able to modulate fibrogenesis in the prolonged Scar-in-a-Jar model as evident by a significant decrease of PRO-C1, PRO-C3, PRO-C6, FBN-C and α-SMA levels in supernatant. In line with the current results, Gao et al. showed that an mTOR inhibitor was able to significantly reduce TGF-β1 induced type III collagen and fibronectin expression within 24 h in primary human lung fibroblasts [23]. Studies using precision-cut IPF lung slices have shown that omipalisib is also capable of modulating the accumulation of PRO-C1 from fibrotic lung tissue in an ex vivo setting [24, 27]. Additionally, a proof of mechanism study showed that treatment of IPF patients with omipalisib for only 7–10 days significantly reduced serum levels of PRO-C3 and PRO-C6, and that these levels correlated with levels of omipalisib in plasma [26]. Nintedanib has been shown to decrease TGF-β1 induced collagen secretion from IPF fibroblasts and non-fibrotic control fibroblasts within 48 h [50]. Nintedanib was also able to prevent an increase in type I collagen mRNA when added to normal fibroblasts cultured for 24 h in an IPF conditioned matrix [49]. These data indicate that mTOR inhibitors and nintedanib have direct effects on the fibroblasts producing collagens and other ECM proteins. Our results indicated that both nintedanib and omipalisib may have cytotoxic effects on the fibroblasts at high concentrations. There was a clear effect on Alamar Blue and partially on LDH release in response to these compounds, indicating a reduction in cell metabolic activity, and a concurrent increase in cytotoxicity, both of which could be beneficial at the correct dose in a fibrotic setting.

In spite of a pronounced effect of the solvent at high concentrations, a reduction of PRO-C1, PRO-C3, PRO-C6 and FBN-C, but not α-SMA, was observed in response to pirfenidone treatment after 8 and 12 days of culture. Interestingly, after 4 days of culture, only PRO-C6 levels were significantly reduced by pirfenidone as compared with vehicle. Pirfenidone at concentrations of 300 and 1000 μM had small but significant cytotoxic effects, as determined by LDH release, as compared to the solvent alone, while metabolic activity assessed by Alamar Blue was not altered. The biomarker data showed no effect on α-SMA assessed in cell supernatant which could indicate that pirfenidone does not alter fibroblast activation but may have a beneficial effect on ECM synthesis. The anti-fibrotic and anti-inflammatory effects of pirfenidone have not been fully established [51]. Some studies have shown that pirfenidone had a direct effect on fibroblasts, whereas others indicated that the effect was very weak [49, 52–55]. However, these studies were performed for a shorter period of 1–2 days which may influence the effect on protein synthesis, as indicated by our results. In a study of IPF fibroblasts, Knüppel et al. found nintedanib to reduce gene expression of type I collagen and fibronectin and attenuate the secretion of type I and III collagen, whereas pirfenidone showed less pronounced effects even in concentrations of up to 1000 μM [48]. Interestingly, both compounds affected type I collagen fibrils by delaying their formation and resulting in fewer and thinner collagen fibrils as compared with untreated controls. Using the same concentration of pirfenidone in the current assay, anti-fibrotic effects of pirfenidone were evident on fibroblasts although effects were more pronounced for nintedanib and omipalisib. When comparing our data with those published by others, it should be taken into consideration that the biomarkers used to assess ECM protein synthesis in the prolonged Scar-in-a-Jar assay are dependent on the release of collagen pro-peptides to the supernatant during protein maturation. Thus, if changes in the amount of collagen that is laid down in the matrix without proper processing occurred, it would not be registered by the biomarkers. Additionally, any effect on fibrosis resolution, which may be enhanced by anti-fibrotic compounds, may not be assessed in this system.

In our experiments we aimed to test anti-fibrotic drugs at concentrations similar to those achieved in patients. After standard dosing of nintedanib, plasma concentrations of approximately 70 nM may be reached [56] and concentrations of 1–1000 nM were used with only minor cytotoxic effects in this model. In the literature, pirfenidone is applied in concentrations up to 10 mM in vitro [54, 57–59] but in an effort to keep within the physiologically relevant range, pirfenidone was applied in concentrations of 30–3000 μM as plasma levels may reach concentrations of 80–100 μM after standard dosing of pirfenidone [56]. Notably, no marked effects were observed for pirfenidone in concentrations below 300 μM. These relatively high concentrations of pirfenidone may force an effect in vitro which is not physiologically relevant. A limitation in our assay is that the higher concentrations of pirfenidone required relatively high concentrations of DMSO which in itself had significant cytotoxic effects at the highest used concentration. However, at concentrations of less than 1000 μM pirfenidone, effects of the solvent were not evident based on the Alamar blue or LDH release.

Even though the investigated in vitro model does not reproduce the complexity of the lung tissue during IPF, it allows for an efficient investigation of the ECM production by lung fibroblasts that resembles the process in vivo, since growing the fibroblast in a ficoll-rich moiety ensures that collagens are deposited in the matrix in the natural conformation [33]. Collagen pro-peptides are cleaved off to allow for correct processing and maturation, including cross-linking. However, a simple single-cell culture which does not include other cell types or production of all relevant proteinases is limited in the investigation of the fibrolytic aspect of collagen turnover. Another limitation of the model is the use of a single pro-fibrotic stimulus, meaning that anti-fibrotic compounds tested in the current model will need to work through the TGF-β1 signaling pathway. Future experiments should investigate the use of other pro-fibrotic mediators, including PDGF, to extend the current model to better represent fibrogenesis. The presented experiments consists of only a few healthy donors of lung fibroblasts, even though the experiment was performed three times to account for technical variability and showed excellent reproducibility. Fibroblasts originating from a range of healthy donors, as well as IPF patients, should be evaluated for their fibrotic response in the prolonged Scar-in-a-Jar model to ensure a continuously working and consistent model.

## Conclusions

In conclusion, the combination of a prolonged Scar-in-a-Jar model with clinically validated biomarkers of ECM synthesis and the evaluation of anti-fibrotic treatments already approved by the FDA, allowed us to develop a robust fibroblast model to use as a screening tool in early drug development for IPF. Additionally, we have shown the importance of the temporal aspect when evaluating ECM accumulation and that more long-term effects should be assessed. This tool may enable the selection of drug candidates and aid translational science, potentially favoring a more successful path in drug development.

## Supplementary information

**Additional file 1:Figure S1.** Effect of pirfenidone shown by day. Lung fibroblasts were stimulated with TGF-β1 and treated with 3 × 10^1^, 1 × 10^2^, 3 × 10^2^ or 1 × 10^3^ μM pirfenidone or corresponding vehicle (0.03, 0.1, 0.3 or 1% DMSO). Biomarkers of ECM synthesis (type I (PRO-C1), III (PRO-C3) and VI (PRO-C6) collagen and fibronectin (FBN-C)) and fibroblast activation (α-SMA) were measured in the supernatant at day 4, 8 and 12. Data are shown as dose-response curves for pirfenidone and its vehicle for the different timepoints and are presented as percentage of the TGF-β1 control over time. All data are shown as mean ± SD of 3 separate experiments each with 4 replicates/treatment and analyzed by two-way ANOVA with Sidak’s multiple comparisons test comparing pirfenidone to vehicle. ns non-significant; *P < 0.05; **P < 0.01; ***P < 0.001; ****P < 0.0001.

## Data Availability

The datasets used and/or analyzed during the current study are available from the corresponding author on reasonable request.

## Abbreviations

ALK5: Activin receptor-like kinase 5
ALK5i: ALK5/type I TGF-β receptor kinase inhibitor
AUC: Area under the curve
BAPN: Beta-aminopropionitrile
DMEM: Dulbecco’s modified eagle medium
DMSO: Dimethyl sulfoxide
ECM: Extracellular matrix
ELISA: Enzyme-linked immunosorbent assay
EMA: European Medicines Agency
FBN-C: ELISA assessing fibronectin
FBS: Fetal bovine serum
FDA: U.S. Food and Drug Administration
IPF: Idiopathic pulmonary fibrosis
LDH: Lactate dehydrogenase
LOX: Lysyl oxidase
mTOR: Mammalian target of rapamycin
PBS: Phosphate-buffered saline
PDGF: Platelet-derived growth factor
PI3K: Phosphatidylinositol 3-kinase
PRO-C1: ELISA assessing type I collagen formation
PRO-C3: ELISA assessing type III collagen formation
PRO-C4: ELISA assessing 7S domain of type IV collagen
PRO-C5: ELISA assessing type V collagen formation
PRO-C6: ELISA assessing type VI collagen formation
PROFILE: Prospective Observation of Fibrosis in the Lung Clinical Endpoints
TGF-β1: Transforming growth factor beta 1
αCTX-1: ELISA assessing cross-linking in the C-terminal telopeptide of type I collagen
α-SMA: alpha-smooth muscle actin
