# Supplementary material for: Prolonged Scar-in-a-Jar: an in vitro screening tool for anti-fibrotic therapies using biomarkers of extracellular matrix synthesis
Source: Respir Res. 2020 May 7;21:108. doi: 10.1186/s12931-020-01369-1 (PMC7203825; doi:10.1186/s12931-020-01369-1)
Supplement: Supplementary file 1 — Additional file 1:Figure S1. Effect of pirfenidone shown by day. Lung fibroblasts were stimulated with TGF-β1 and treated with 3 × 101, 1 × 10^2^, 3 × 10^2^ or 1 × 10^3^ μM pirfenidone or corresponding vehicle (0.03, 0.1, 0.3 or 1% DMSO). Biomarkers of ECM synthesis (type I (PRO-C1), III (PRO-C3) and VI (PRO-C6) collagen and fibronectin (FBN-C)) and fibroblast activation (α-SMA) were measured in the supernatant at day 4, 8 and 12. Data are shown as dose-response curves for pirfenidone and its vehicle for the different timepoints and are presented as percentage of the TGF-β1 control over time. All data are shown as mean ± SD of 3 separate experiments each with 4 replicates/treatment and analyzed by two-way ANOVA with Sidak’s multiple comparisons test comparing pirfenidone to vehicle. ns non-significant; *P < 0.05; **P < 0.01; ***P < 0.001; ****P < 0.0001. [file 12931_2020_1369_MOESM1_ESM.pdf]

Supplementary material for “Prolonged Scar-in-a-Jar: an *in vitro* screening tool for anti-fibrotic therapies using biomarkers of extracellular matrix synthesis”

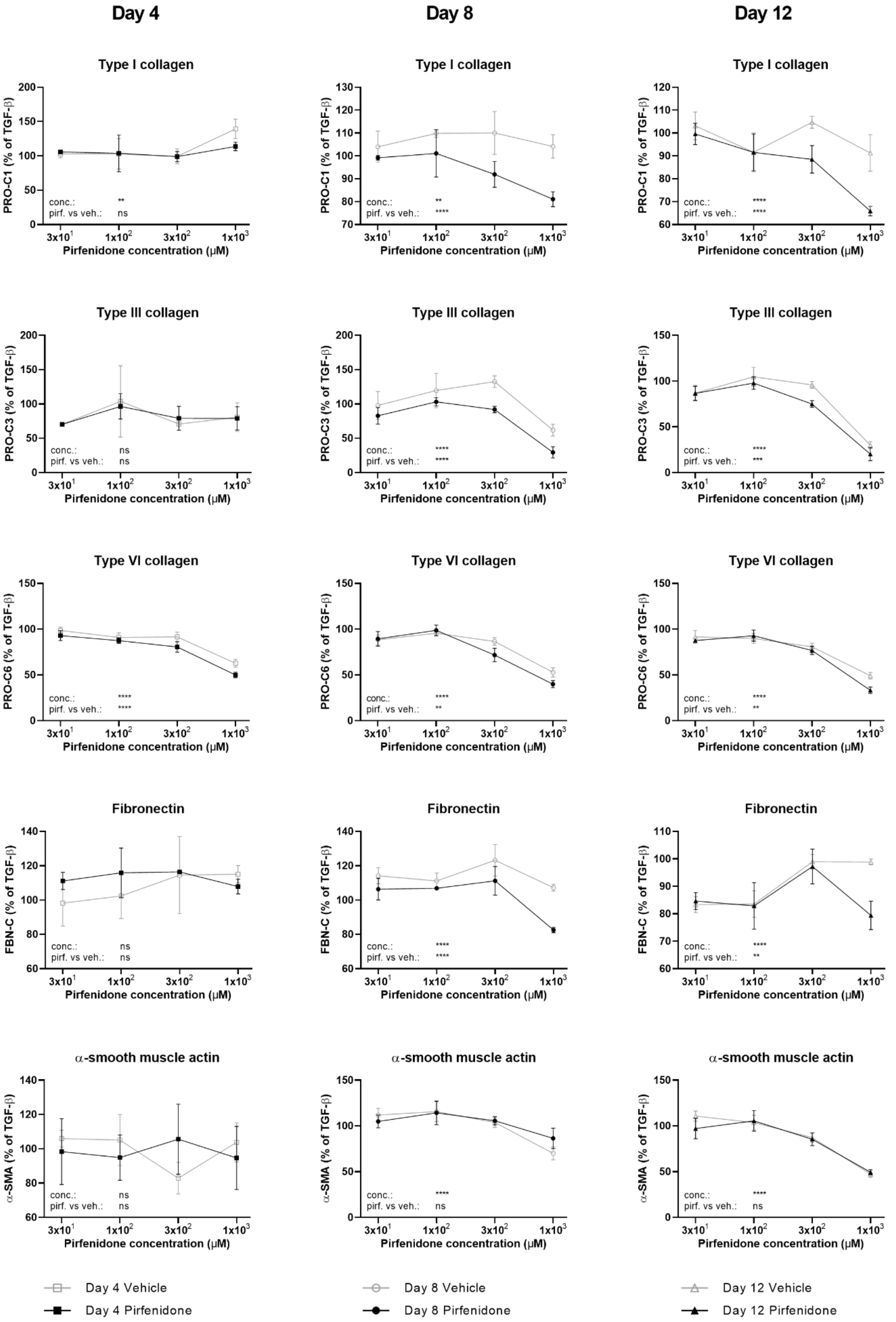

Supplementary figure 1: Effect of pirfenidone shown by day

Lung fibroblasts were stimulated with TGF-β1 and treated with $3x10^1$, $1x10^2$, $3x10^2$ or $1x10^3$ µM pirfenidone or corresponding vehicle (0.03%, 0.1%, 0.3% or 1% DMSO). Biomarkers of ECM synthesis (type I (PRO-C1), III (PRO-C3) and VI (PRO-C6) collagen and fibronectin (FBN-C)) and fibroblast activation (α-SMA) were measured in the supernatant at day 4, 8 and 12. Data are shown as dose-response curves for pirfenidone and its vehicle for the different timepoints and are presented as percentage of the TGF-β1 control over time. All data are shown as mean±SD of 3 separate experiments each with 4 replicates/treatment and analyzed by two-way ANOVA with Sidak's multiple comparisons test comparing pirfenidone to vehicle. ns non-significant; *P<0.05; **P<0.01; ***P<0.001; ****P<0.0001.
